# Transcutaneous Auricular Vagus Nerve Stimulation at 20 Hz Improves Depression-Like Behaviors and Down-Regulates the Hyperactivity of HPA Axis in Chronic Unpredictable Mild Stress Model Rats

**DOI:** 10.3389/fnins.2020.00680

**Published:** 2020-07-15

**Authors:** Shaoyuan Li, Yu Wang, Guojian Gao, Xiao Guo, Yue Zhang, Zixuan Zhang, Yifei Wang, Jinling Zhang, Junying Wang, Liang Li, Yongsheng Yang, Peijing Rong

**Affiliations:** ^1^Institute of Acupuncture and Moxibustion, China Academy of Chinese Medical Sciences, Beijing, China; ^2^China Academy of Chinese Medical Sciences, Beijing, China

**Keywords:** transcutaneous auricular vagus nerve stimulation (taVNS), frequency, depressive-like behavior, hypothalamic-pituitary-adrenal (HPA) axis, chronic unpredictable mild stress (CUMS)

## Abstract

Transcutaneous auricular vagus nerve stimulation (taVNS) has gained growing interest as a non-invasive and non-pharmacologic treatment option in various neurological and psychiatric disorders. Animal experiments and clinical trials confirm that taVNS at the auricular concha region has beneficial effects on depression. However, stimulation frequencies are selected empirically, and there is no evidence showing that any frequency is superior to the others. This study explores antidepressant-like effects of three frequencies of taVNS on rats subjected to chronic unpredictable mild stress (CUMS). Sprague-Dawley rats were randomly divided into five groups, i.e., the control, CUMS, 5 Hz-taVNS, 20 Hz-taVNS, and 100 Hz-taVNS groups. The three different frequencies were administered during the 30-min taVNS procedure once a day for 28 consecutive days. Rats exposed to CUMS showed signs of depression-like behaviors, including reduction in sucrose preference and increased immobility time in forced swimming and open field tests as well as significant dysfunction of the hypothalamic-pituitary-adrenal (HPA) axis as detected by plasma corticosterone and adrenocorticotropic hormone concentration. The 28 days’ taVNS sessions with three frequencies elicited quite different consequences. Although 20 Hz taVNS significantly reversed the depression-like behaviors and downregulated the hyperactivity of the HPA axis, neither 5 nor 100 Hz showed any antidepressant-like effect on CUMS-induced rat behavior. Based on these results, we propose that, out of the three frequencies for taVNS intervention on depression, 20 Hz may be the optimized frequency to have a better modulation effect on HPA axis function by activating the auricular vagus nerve.

## Introduction

Depression is a frequent and prevalent clinical complaint. In 2015, the proportion of the global population with depression was estimated to be 4.4% (World Health Organization, 2017). People with depressive disorders are disturbed by sadness, interest or pleasure loss, guilt or low self-worth feelings, sleep (Chan et al., 2014) or appetite disturbance (Simmons et al., 2016), and poor concentration. Moreover, long-lasting or recurrent depression may substantially impair the ability to cope with work or daily life and can even lead to suicide. Despite recent advances in antidepressants, patients suffer from a variety of problems, such as adverse effects and no response to medicine (Bull et al., 2002; Racagni and Popoli, 2010; Segraves and Balon, 2014).

As a slow-acting therapy, cervical vagus nerve stimulation (VNS) was approved by the United States Food and Drug Administration (FDA) for chronic treatment-resistant depression in 2005 (Yuan and Silberstein, 2016). As of June 2018, more than 100,000 patients have received VNS therapy worldwide (Wheless et al., 2018). It is reported that auricular concha is the only area on the body surface that is distributed with ear vagus nerve fiber endings (Peuker and Filler, 2002). Inspired by the mechanism of VNS, we and other researchers have proven that transcutaneous auricular vagus nerve stimulation (taVNS), a non-invasive method, also has a curative effect on depression (Rong et al., 2016; Wu et al., 2018). Additionally, the brain loci activated by VNS and taVNS are coincident (Badran et al., 2018). It is assumed that stimulation on the auricular vagus nerve can activate the inferior ganglion, which projects to the nucleus tractus solitarii (NTS) (He et al., 2013) and affects the activities of depression-related cortical-limbic-thalamic neural circuits through connections between NTS and other brain regions, such as the locus coeruleus, parabrachial nucleus, hypothalamus, thalamus, amygdala, hippocampus, anterior cingulate cortex, anterior insula, and lateral prefrontal cortex (Beekwilder and Beems, 2010). Thus, the vagus nerve has direct and indirect connections to the depression-related cortical-limbic-thalamic neural circuits, influencing the activities of these regions (Kosel et al., 2011; Ruffoli et al., 2011). Previous studies have exhibited that taVNS is an effective treatment for depression (Hein et al., 2013; Rong et al., 2016), which significantly modulates the default mode network functional connectivity in patients with mild or moderate major depressive disorder (MDD) (Fang et al., 2016).

Frequency is a crucial parameter of nerve electrical stimulation. It has been confirmed that different chemical substances from the brain can be activated and produced by different frequency electrical current stimulation. It has been proven that electro-stimulation at 2 and 100 Hz can promote the production of enkephalin and dynorphin in the brain, respectively, with a corresponding relationship (Han, 2003). It is also a typical example of the principle of traditional electrical stimulation supported by modern medical evidence. The frequency specificity of electroacupuncture has been confirmed gradually in the treatment of pain, detoxification, autism, infertility, and other diseases, and the optimal frequency of electroacupuncture corresponding to different diseases is also different, showing frequency specificity (Han, 2003).

Previous studies used a wide range of frequencies (Zhang et al., 2003; Hein et al., 2013; Rong et al., 2016) of taVNS in the treatment of depression, but few of them explored the optimal frequency of taVNS on depression. Therefore, we conducted an animal experiment to explore the antidepressant effects of three taVNS frequencies (5, 20, and 100 Hz) by comparing behavioral changes (assessed by sucrose water preference rate, forced swimming immobility time, and open-field movement score) and function of hypothalamus-pituitary-adrenal (HPA) axis [assessed by plasma corticosterone (CORT) and adrenocorticotropic hormone (ACTH) concentrations] of chronic unpredictable mild stress (CUMS) depression model rats.

## Materials and Methods

### Animals

**FIGURE 1:**
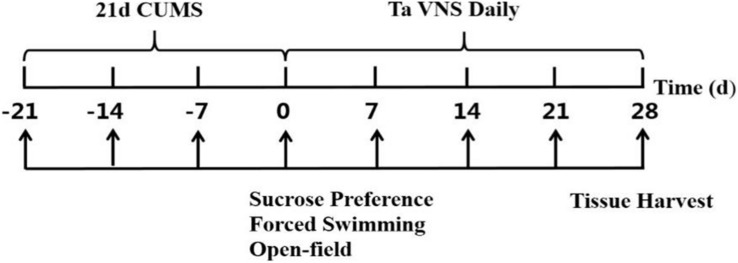
Illustration of the experiment design.

Fifty male Sprague-Dawley rats weighing 180–230 g at 6 weeks of age were purchased from the Laboratory Animal Center of the Academy of Military Medical Sciences [Certificate no. SCXK (jing) 2014-0013]. The rats were single-housed at temperatures of 22°C ± 1°C and humidity of 50% ± 5%. Except during CUMS and taVNS procedures, animals were housed with free access to purified water and standard chow. The rats were randomly divided into five groups, i.e., the control, CUMS, 5 Hz-taVNS, 20 Hz-taVNS, and 100 Hz-taVNS groups (*n* = 10 per group). Except the control group, all other groups underwent the CUMS procedure for 21 days. Afterward, the CUMS rats in the taVNS-treated groups additionally received daily electrical stimulation at frequencies of 5, 20, and 100 Hz, respectively, for the following 28 days. All animals were decapitated, and blood samples were collected at the end of experiment procedure on day 28. The overall design is shown in Figure 1. This study was carried out in strict accordance with the recommendations in the Guidelines for the Care and Use of Laboratory Animals of the National Institute of Health. The protocol of the experiment was approved by Animal Care and Use Committee of the Institute of Acupuncture and Moxibustion, China Academy of Chinese Medical Sciences (D2017-08-16-1). All efforts were made to minimize the suffering.

### Chronic Unpredictable Mild Stress (CUMS)

Except the control group, all animals were exposed to CUMS combined with isolation for 21 days to induce depression-like behavior. According to Willner (2017), we used seven mild stressors: 5 min ice water swimming, 12 h day and night inversion, 24 h wet pecking, 1 min tail clipping, 5 min hot plate (52°C, Ugo Basile s.r.l. hot/cold plate, Model 35100–001, Italy), 24 h food deprivation, and 24 h water deprivation. Each stressor was conducted randomly but not repetitively every day in order to avoid the prediction of animals.

### Administration of taVNS

After 21 days of the CUMS procedure, the three experimental groups received taVNS at the frequency of 5, 20, and 100 Hz, respectively, by an electrical stimulator (Hua tuo SDZ-IIB, Suzhou, China) each day for 28 consecutive days. Under 2% isoflurane inhalation anesthesia (Matrx VIP 3000, Midmark Corporation, United States), the positive and negative electrodes were placed at the rats’ ear over the skin inside and outside of the rats’ auricular concha region to ensure the pass of the electric current. Other taVNS parameters used for the three experimental groups were the same (2 mA intensity, 30 min per day) and were already proven effective by our previous studies (Zhai et al., 2016). The taVNS procedure was administrated daily at the same time between 9 and 11 am to weaken the influence of biological rhythm (Li et al., 2014).

### Behavioral Tests

To record stress-induced, depression-like behavior, sucrose preference (SP), forced swimming (FST), and open-field tests (OFT) were measured once a week. All tests were conducted between 7 and 9 am before taVNS. Experiments were performed in a special behavioral testing room in which animals were not housed by the same persons blind to the allocation of treatment.

Sucrose preference (SP) is considered to be associated with anhedonia (Forbes et al., 1996). To habituate the rats, two identical bottles containing 1% sucrose were provided for 24 h before the first preference test followed by a 23-h fluid deprivation interval at day −21 (Figure 1). Afterward, two bottles with sucrose solution and water were offered for 1 h, respectively, to determine the baseline. SP was calculated as the percentage of sucrose solution consumption per total fluid consumption.

Forced swimming (FST) was performed with slight modifications according to Porsolt et al. (1977). Each rat was placed in a transparent plastic tank of 45 × 35 × 60 (width × length × height in cm) filled up to a height of 30 cm with water (20°C–25°C) for 5 min. After the first minute, the immobile time, in which the rat moves only to keep the head above water, was recorded for 3 min. Afterward, rats were removed and wiped dry. The tank was cleaned after each rat. It was noticed that the water temperature was crucial for the test since either too high or too low temperature could affect the animal immobility time (Porsolt et al., 1977).

Open-field tests (OFT) is predicated on the rat’s natural drive to explore new areas and the immanent anxiety toward them and usually measures vertical (locomotion) and horizontal (rearing) movements (Stanford, 2007). Each rat was placed in the center of an open box of 80 × 80 × 40 cm (width × length × height) with a black floor and walls. The floor was divided into 25 squares. The rat was allowed to explore the area for 5 min. Movements were videorecorded. Crossing grid line scores, referring to general locomotor activity during exploration, and rearing scores (when both frontal paws were uplifted) were counted after the first minute for 3 min (Willner, 2017). The box was cleaned by use of ethanol after each trial.

### Plasma Collection and ELISA

All animals were sacrificed, and the neck venous blood was collected at the same time by the end of the whole experiment. After centrifugation, samples were stored at −80°C until enzyme-linked immunosorbent assay (ELISA) for CORT (BlueGene, E02C0006, China) and ACTH (BlueGene, E02A0005, 201702, China) were performed according to the manufacturer’s protocol and analyzed using a microplate reader (Multiskan MK3, Thermo Scientific, Beijing, China).

### Statistical Analysis

Sucrose preference (SP), Forced swimming (FST), and Open-field tests (OFT) were analyzed using repeated-measures analysis of variance (ANOVA; time effect) with conditioning effects. Time effects corresponded to the effects of measure repetition (i.e., −21, −14, −7, 0, 14, 21, and 28 days). Group effects corresponded to the effects of group classification (i.e., control, CUMS, 5 Hz, 20 Hz, and 100 Hz groups). Group*Time effects corresponded to the combined effects of the time and the group classification. When ANOVA revealed a significant effect, a *post hoc* Bonferroni test for all couples were carried out. ELISA results were compared using one-way ANOVA to detect differences among treatment groups, followed by the Tukey-Kramer multiple comparisons test to determine sources of differences. The significance level was set at *P* < 0.05. Data are presented as mean ± standard deviation.

## Results

### Effect of Different Frequencies of taVNS on Sucrose Preference

**FIGURE 2:**
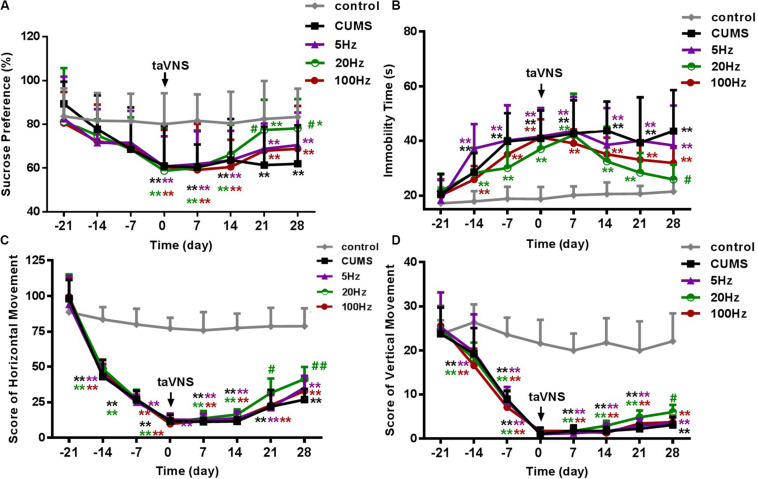
Different frequencies of taVNS on behavior of CUMS rats. **(A,B)** show the comparison of sucrose preference and immobility time of FST, and **(C,D)** are a comparison of horizontal and vertical movement score, two contents of the open-field test, among different groups of the same abscissa point, respectively. *, **P < 0.05, *P* < 0.01 vs. control group and ^#^*P* < 0.05, ^##^*P* < 0.01 vs. CUMS group.

There was no statistical difference in SP among the groups at the baseline (-21 day). During the 21 days of the CUMS procedure without treatment, a loss in sucrose preference as a sign of depression-like behavior in rats was observed in the CUMS and CUMS with 5 Hz-taVNS, 20 Hz-taVNS, and 100 Hz-taVNS groups compared with the non-stressed control group. Starting with taVNS treatment from day 0, sucrose preference significantly increased until days 21 and 28 only in the 20 Hz group (*P* < 0.05 and *P* < 0.01) compared to other CUMS groups but was still below that of the control group. No statistical difference was found among the CUMS and CUMS with 5 Hz-taVNS and 100 Hz-taVNS groups (Figure 2A).

### Effect of Different Frequencies of taVNS on Immobility Time in the FST and on Open-Field Test Score

On day −21, the immobility time of the different groups was not different. After 21 days of CUMS, the immobility time of the stressed groups increased compared to the control group (*P* < 0.01). However, exclusively taVNS treatment with 20 Hz, but not with 5 or 100 Hz for 28 days improved the behavior in the FST (*P* < 0.01) (Figure 2B).

Similar to the FST, horizontal and vertical movements in the open-field test did not differ between the groups before introduction of the CUMS protocol but significantly decreased within 21 days in all CUMS groups (*P* < 0.01). Intervention with 20 Hz-taVNS significantly increased both the horizontal and vertical movements compared with those of the CUMS group (*P* < 0.01). In particular, an increase in horizontal movements was observed on day 21 (*P* < 0.05), earlier than that of vertical movements (*P* < 0.05 on day 28). No significant alterations were found in the open field behavior of the 5 and 100 Hz-taVNS groups compared with the untreated CUMS group (Figures 2C,D). It should be noted that even the CUMS group exhibited a tendency to an enhanced number of horizontal crossings at the end of the experiment (Figure 2C), indicating that the CUMS depression model may have a potential of spontaneous recovery without any intervention.

### Effect of Different Frequencies of taVNS on Plasma CORT and ACTH

**FIGURE 3:**
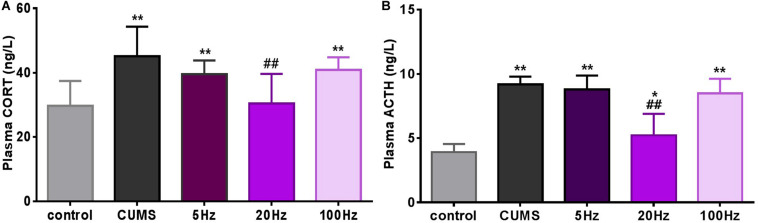
Different frequencies of taVNS plasma CORT and ACTH concentration of CUMS rats. *, **P < 0.05, *P* < 0.01 vs. control group and ^#^*P* < 0.05, ^##^*P* < 0.01 vs. CUMS group.

Stress hormones were analyzed 28 days after introduction of taVNS-treatment. CUMS exposure significantly affected plasma concentration of CORT (*P* < 0.01) compared to controls. A decreasing trend of the mean concentration of plasma CORT was observed in the CUMS group and different frequencies taVNS groups exposed to CUMS, but a significant difference was only found between the 20-Hz group and the CUMS group (*P* < 0.01) (Figure 3A).

Similarly, plasma concentrations of ACTH in the CUMS groups were significantly increased compared to the controls (*P* < 0.05 for the 20 Hz-taVNS group and *P* < 0.01 for the others). Only the treatment with 20 Hz-taVNS caused a significant reduction of the stress-induced high concentration of ACTH in the CUMS group (*P* < 0.01) (Figure 3B).

## Discussion

In general, rats exposed to 21 days of CUMS showed depression-like behaviors measured by SP, FST, and OFT. taVNS intervention at a frequency of 20 Hz could improve the abnormal behaviors and keep plasma concentration of CORT and ACTH at a lower level than taVNS at 5 and 100 Hz.

### Frequency Is a Crucial Parameter in taVNS

Frequency is an important parameter for various forms of physical stimulation including taVNS, which can directly affect the curative efficacy of the interventions (Aaronson et al., 2013; Xie et al., 2013; Iaccarino et al., 2016). The range of taVNS frequencies used in previous studies for depression is wide. Hein et al. (2013) reported that 1.5 Hz unipolar rectangle waves significantly decreased the Beck Depression Inventory (BDI) score but not Hamilton Depression Rating Scale (HAMD) score. Our team found that a 20 Hz continuous sinusoidal wave made significant improvement compared with sham treatment in more commonly used depression scales, such as HAMD/SDS, SAS, and HAMA (Rong et al., 2016). For the potential mechanism, previous studies indicated that different frequencies of electrical stimuli could produce different brain changes and neurotransmitter releases (Han, 2003; Zhang et al., 2003). In an animal study (Wang et al., 2012), investigators explored the anti-epileptic effect of taVNS with different stimulation frequencies and durations in rats and found that taVNS at 20 Hz had a significantly stronger anti-epileptic effect t taVNS at 2 and 100 Hz as measured by the duration of seizure suppression. Consistent with the above results, a recent clinical study (Bauer et al., 2016) on taVNS treatment of drug-resistant epilepsy showed a significant reduction in seizure frequency in patients of the 25 Hz group compared to the 1 Hz group. Taken together, these studies imply that the optimal stimulation frequency might activate the central nervous system, in particular the central autonomic network depending on the type of disorder.

Additionally, the common frequency of brain waves ranges from 1 to 30 Hz, such as theta (4–12 Hz), delta (1–4 Hz), and beta (12–30 Hz), which has been examined to study emotional states recently. Chronic VNS has a regulatory action via afferent vagal fibers on anxiodepressive symptomatology, which could be directly highlighted by the modulation of EEG associated to depressed states (Kibleur et al., 2018). It is hypothesized that beta frequency is closely associated with negative mood, stress, and depression (Huiku et al., 2007; Li et al., 2016). Twenty Hz, in the range of beta frequency, may be adapted to the right wave frequency, which can modulate the abnormal activity of brain waves in depressive disorder. This phenomenon may provide a possible interpretation for the results that taVNS at 20 Hz produced a better result compared with taVNS at 5 and 100 Hz.

### 20 Hz taVNS Improved Depressive Status in Behavioral Tests of CUMS Rats

Chronic unpredictable mild stress (CUMS) has long been used in the study of depression and is considered to be particularly relevant to human depression (Tanti and Belzung, 2010; Paul, 2016). This model was first adopted in a series of studies by Katz and colleagues in the 1980s (Katz and Hersch, 1981; Katz, 1982) and now has made a significant contribution to studies of the neurobiological mechanism of depression and the efficacy of clinical antidepressant measures, especially the crucial role of the HPA axis. The validity and translational potential of animal models of depression disorders are usually assessed with a multidimensional approach, such as SP, FST, and OFT. SP is a valid measure of sensitivity to reward and anhedonia (Strekalova et al., 2004), the clinical core symptom of depression, which is a main behavioral index on the depressive-like model. SP can be decreased generally by CUMS. The immobility time of FST and score of OFT are two auxiliary indices, which are also used in many studies of depression, which is recognized as an expression of despair (Prut and Belzung, 2003). In this study, 28 days of taVNS at 20 Hz significantly improved these depressive-like behaviors, but no significant improvement was found in the 5 Hz-taVNS or 100 Hz-taVNS groups. These results indicated that taVNS was effective in relieving depressive-like behaviors of CUMS rats, and 20 Hz was a better choice compared with high and low frequency. Some studies had no doubt about the effects of acupuncture on these indicators in depression models although the researchers chose lower frequencies (2 or 5 Hz); different areas and acupoints of stimulation or combining with other antidepressant agents may explain the different results of preferable frequency (Yu et al., 2007; Yang et al., 2013).

Indeed, compared with invasive VNS to activate vagal efferent of the cervical vagus nerve, 20 Hz-taVNS can better activate the afferents than 5 Hz. And 100 Hz may damage the vagus nerve because of the high frequency. Moreover, the results of this study are consistent with our previous research on human beings with MDD in which the frequency of taVNS that we chose was 20 Hz (Fang et al., 2016; Rong et al., 2016). The basic experiment may just be the corroborative evidence of previous clinical contribution.

### 20 Hz taVNS Modulated the Hyperfunction of the HPA Axis of CUMS Rats

The major stress-responsive system is the HPA axis, which is one of the most important neuroendocrine axes in our human body, which is widely proven to be closely related to pathologic mechanism of depression (Herman et al., 2003; Barden, 2004; Liyanarachchi et al., 2017). Whether alteration in the HPA axis leads to depression or a consequence of depression is still under argument (Holsboer and Barden, 1996; Nemeroff and Vale, 2005). Main components produced and released from the HPA axis include corticotropin-releasing hormone (CRH), ACTH, and CORT. Stress, including psychogenic and physiological as well as other irritant factors, stimulate the paraventricular nucleus of the hypothalamus to release CRH. By transportation through the hypophyseal portal system, CRH promotes secretion of ACTH, which activates growth of the adrenal cortex-zona fasciculata and reticularis to stimulate CORT secreting into systemic circulation. When plasma CORT increases beyond the normal concentration, excessive amounts of CORT combine with relevant receptors to initiate negative feedback to the HPA axis, which can induce the decline of CRH and ACTH secretion as well as CORT. The most important CORT receptor mainly distributes in the hippocampus, which plays a crucial role in emotion regulation, learning, and memory ability. Moreover, a large amount of clinical research and animal experiments demonstrate that the negative feedback mechanism of the HPA axis is impaired in depression. The disfunction of the HPA axis caused by long periods of stress induces abnormal secretion of CORT in saliva, cerebrospinal fluid, urine, and plasma. Hippocampal atrophia aggravates due to the continuous elevation of CORT concentration in stressed rats, inversely, volume will recover with the decrease of CORT when an adrenalectomy is performed (Gould et al., 1992). The mechanism of the clinical efficacy of some antidepressants may be interpreted by upregulating the glucocorticoid and mineralocorticoid receptors in the brain (de Kloet et al., 2007).

In this study, high levels of circulating CORT and ACTH can be seen in rats exposed to 21 days’ CUMS, indicating hyperfunction of the HPA axis in CUMS rats. Twenty Hz taVNS intervention relieved the depressive status, which indicates that the antidepressive effect of 20 Hz taVNS might have the potential effect to modulate HPA axis function by activating the auricular vagus nerve, which led to the reversion of plasma CORT and ACTH concentrations. Twenty Hz may be an optimized frequency of taVNS on depression. Further investigation shall be warranted for this hypothesis.

### Limitations and Future Directions

There are two main limitations of this study. First, we only explore the different frequencies of taVNS in an animal model of stress-induced depression. Other factors, such as intensity, duration, and more accurate divisions on frequency of treatment, all play crucial roles in the evaluation of effect, which calls for further systematic research on this topic. Second, the mechanism behind the taVNS frequency is not clear; more brain loci and indexes should be focused on in the future.

## Conclusion

Chronic unpredictable mild stress rats show depression-like behaviors and hyperfunction of the HPA axis. taVNS at 20 Hz frequency seems more effective in reversing behavioral symptoms of anhedonia (SPT) and passive coping strategies (FST) than anxiety-like symptoms (OFT) in CUMS-exposed rats through downregulating hyperactivity of the HPA axis on the CUMS rats.

## Data Availability Statement

The raw data supporting the conclusions of this article will be made available by the authors, without undue reservation, to any qualified researcher.

## Ethics Statement

The animal study was reviewed and approved by the Ethics Committee, Institute of Acupuncture and Moxibustion, China Academy of Chinese Medical Sciences.

## Author Contributions

PR and YY designed the protocol. SL, GG, XG, YZ, ZZ, and JZ performed the experiments. SL, YW, and YW analyzed the data. SL, JW, LL, and PR wrote the manuscript. All authors contributed to the article and approved the submitted version.

## Conflict of Interest

The authors declare that the research was conducted in the absence of any commercial or financial relationships that could be construed as a potential conflict of interest.
